# Complete Genome Sequence of Acidithiobacillus ferridurans JCM 18981

**DOI:** 10.1128/MRA.01028-18

**Published:** 2018-08-23

**Authors:** Tomoko Miyauchi, Atsushi Kouzuma, Takashi Abe, Kazuya Watanabe

**Affiliations:** a School of Life Sciences, Tokyo University of Pharmacy and Life Sciences, Horinouchi, Hachioji, Tokyo, Japan; b Graduate School of Science and Technology, Niigata University, Niigata, Niigata, Japan; Queens College

## Abstract

Acidithiobacillus ferridurans is an acidophilic chemolithotrophic bacterium that can grow in the presence of high concentrations of ferrous iron. Here, we present the complete 2,921,399-bp genome sequence of the strain A. ferridurans JCM 18981^T^, isolated from uranium mine drainage water.

## ANNOUNCEMENT

The genus Acidithiobacillus includes acidophilic chemolithotrophs that obtain energy for growth by oxidizing inorganic compounds, such as ferrous iron, elemental sulfur, reduced sulfur compounds, and hydrogen (1). Members of this genus have attracted attention due to their potential uses in bioleaching processes (2) and microbial electrosynthesis systems (3). Acidithiobacillus ferridurans is a recently described species that was formerly incorrectly identified as Acidithiobacillus ferrooxidans (4). The type strain of A. ferridurans is JCM 18981^T^ (=ATCC 33020^T^), which was originally isolated in 1976 from drainage water at a uranium mine in Japan (5). Compared with other strains of Acidithiobacillus, this strain exhibits high tolerance to ferrous iron (4) and superior growth with hydrogen (6).

A. ferridurans JCM 18981^T^ was obtained from the Japan Collection of Microorganisms (JCM) and was grown at 30°C in sealed vials containing a mineral salts medium (7) and carbon dioxide, hydrogen, and oxygen as the carbon source, electron donor, and electron acceptor, respectively. Genomic DNA was extracted using the standard cetyltrimethylammonium bromide (CTAB) protocol (8) and sequenced using the PacBio RS II platform (Pacific Biosciences, Menlo Park, CA, USA). The single-molecule real-time (SMRT) cell 8Pac version 3 and DNA polymerase binding kit P6 (Pacific Biosciences) were used for library preparation. Sequencing generated 107,470 filtered subreads (1,020,712,522 bp) with an average length of 9,497 bp. These reads were assembled into a single circular contig with an average coverage of 283 using the Hierarchical Genome Assembly Process version 3.0 (Pacific Biosciences), and the assembly was annotated using Prokka version 1.12b (9). All software was used with default parameters.

The complete genome sequence of A. ferridurans JCM 18981^T^ comprises a 2,921,399-bp chromosome with a GC content of 58.4%. Genome analysis identified 3,026 protein-coding sequences (CDSs), 47 tRNA genes, and 6 rRNA operons. The functional annotation of the CDSs revealed the presence of genes for the Calvin-Benson-Bassham cycle (e.g., types I and II ribulose-1,5-bisphosphate carboxylase/oxygenase genes), nitrogen fixation (genes for nitrogenase NifHDK and associated proteins), and iron oxidation (the *rus* and *petI* operons). Genes encoding four types of hydrogenases present in A. ferrooxidans ATCC 23270^T^ (genes for the groups 1, 2, 3b, and 4 hydrogenases) (2) were also found in this strain, which support its ability to utilize hydrogen. The use of hydrogen as an electron donor is meritorious for Acidithiobacillus strains because it can conserve more energy than with the oxidation of ferrous iron (6). In addition, it does not form toxic precipitates, such as ferric oxides, that are inhibitory for cell growth and problematic for subsequent experiments, including nucleic acid extraction. We therefore suggest that A. ferridurans JCM 18981^T^ is useful as a model organism for investigating genetic and physiological features of acidophilic chemolithotrophs owing to its ease of cultivation, experimental manipulation, and annotated genomic sequence. We also expect that comparative genomics among Acidithiobacillus strains, including A. ferridurans JCM 18981^T^, A. ferrooxidans ATCC 23270^T^, A. ferrivorans SS3, and A. caldus ATCC 51756^T^, will identify genetic backgrounds for physiological characteristics common in this genus and those peculiar to each species.

### Data availability.

The complete genome sequence has been deposited at DDBJ/GenBank under the accession number AP018795. The version described in this paper is the first version, AP018795.1.
